# Supplementary material for: Resolving the full spectrum of human genome variation using Linked-Reads
Source: Genome Res. 2019 Apr;29(4):635–45. doi: 10.1101/gr.234443.118 (PMC6442396; doi:10.1101/gr.234443.118)
Supplement: Supplemental Material [file supp_gr.234443.118_Supplemental_longranger-2.2.zip › longranger-2.2.tar.bz2!/lr2.2-public/lib/stan/cmdstan/stan/doc/api/html/inv__wishart_8hpp_source.html]

Stan: src/stan/prob/distributions/multivariate/continuous/inv\_wishart.hpp Source File


|  |  |
| --- | --- |
|  | Stan  2.5.0  probability, sampling & optimization |


- Main Page
- Related Pages
- Namespaces
- Classes
- Files

- File List
- File Members

All Classes Namespaces Files Functions Variables Typedefs Enumerations Enumerator Friends Macros Pages

- src
- stan
- prob
- distributions
- multivariate
- continuous

inv\_wishart.hpp

Go to the documentation of this file.

1 #ifndef STAN\_\_PROB\_\_DISTRIBUTIONS\_\_MULTIVARIATE\_\_CONTINUOUS\_\_INV\_WISHART\_HPP

2 #define STAN\_\_PROB\_\_DISTRIBUTIONS\_\_MULTIVARIATE\_\_CONTINUOUS\_\_INV\_WISHART\_HPP

3

4 #include <stan/agrad/rev.hpp>

5 #include <stan/agrad/rev/matrix.hpp>

6 #include <stan/math/error\_handling/matrix/check\_ldlt\_factor.hpp>

7 #include <stan/math/error\_handling/check\_greater.hpp>

8 #include <stan/math/error\_handling/matrix/check\_size\_match.hpp>

9 #include <stan/math/matrix/meta/index\_type.hpp>

10 #include <stan/math/matrix/log\_determinant\_ldlt.hpp>

11 #include <stan/math/matrix/mdivide\_left\_ldlt.hpp>

12 #include <stan/meta/traits.hpp>

13 #include <stan/prob/constants.hpp>

14 #include <stan/prob/traits.hpp>

15 #include <stan/prob/distributions/multivariate/continuous/wishart.hpp>

16

17 namespace stan {

18  namespace prob {

19  // InvWishart(Sigma|n,Omega) [W, S symmetric, non-neg, definite;

20  // W.dims() = S.dims();

21  // n > S.rows() - 1]

49  template <bool propto,

50  typename T\_y, typename T\_dof, typename T\_scale>

51  typename boost::math::tools::promote\_args<T\_y,T\_dof,T\_scale>::type

52  inv\_wishart\_log(const Eigen::Matrix<T\_y,Eigen::Dynamic,Eigen::Dynamic>& W,

53  const T\_dof& nu,

54  const Eigen::Matrix<T\_scale,Eigen::Dynamic,Eigen::Dynamic>& S) {

55  static const char\* function = "stan::prob::inv\_wishart\_log(%1%)";

56

57  using boost::math::tools::promote\_args;

58  using Eigen::Dynamic;

59  using Eigen::Matrix;

60  using stan::math::check\_greater;

61  using stan::math::check\_size\_match;

62  using stan::math::index\_type;

63

64  typename index\_type<Matrix<T\_scale,Dynamic,Dynamic> >::type k

65  = S.rows();

66  typename promote\_args<T\_y,T\_dof,T\_scale>::type lp(0.0);

67

68  check\_greater(function, nu, k-1, "Degrees of freedom parameter",

69  &lp);

70  check\_size\_match(function,

71  W.rows(), "Rows of random variable",

72  W.cols(), "columns of random variable",

73  &lp);

74  check\_size\_match(function,

75  S.rows(), "Rows of scale parameter",

76  S.cols(), "columns of scale parameter",

77  &lp);

78  check\_size\_match(function,

79  W.rows(), "Rows of random variable",

80  S.rows(), "columns of scale parameter",

81  &lp);

82

83  // FIXME: domain checks

84

85  using stan::math::lmgamma;

86  using stan::math::log\_determinant\_ldlt;

87  using stan::math::mdivide\_left\_ldlt;

88  using stan::math::trace;

89  using stan::math::LDLT\_factor;

90  using stan::math::check\_ldlt\_factor;

91

92  LDLT\_factor<T\_y,Eigen::Dynamic,Eigen::Dynamic> ldlt\_W(W);

93  check\_ldlt\_factor(function,ldlt\_W,"LDLT\_Factor of random variable",&lp);

94  LDLT\_factor<T\_scale,Eigen::Dynamic,Eigen::Dynamic> ldlt\_S(S);

95  check\_ldlt\_factor(function,ldlt\_S,"LDLT\_Factor of scale parameter",&lp);

96

97  if (include\_summand<propto,T\_dof>::value)

98  lp -= lmgamma(k, 0.5 \* nu);

99  if (include\_summand<propto,T\_dof,T\_scale>::value) {

100  lp += 0.5 \* nu \* log\_determinant\_ldlt(ldlt\_S);

101  }

102  if (include\_summand<propto,T\_y,T\_dof,T\_scale>::value) {

103  lp -= 0.5 \* (nu + k + 1.0) \* log\_determinant\_ldlt(ldlt\_W);

104  }

105  if (include\_summand<propto,T\_y,T\_scale>::value) {

106 // L = crossprod(mdivide\_left\_tri\_low(L));

107 // Eigen::Matrix<T\_y,Eigen::Dynamic,1> W\_inv\_vec = Eigen::Map<

108 // const Eigen::Matrix<T\_y,Eigen::Dynamic,Eigen::Dynamic> >(

109 // &L(0), L.size(), 1);

110 // Eigen::Matrix<T\_scale,Eigen::Dynamic,1> S\_vec = Eigen::Map<

111 // const Eigen::Matrix<T\_scale,Eigen::Dynamic,Eigen::Dynamic> >(

112 // &S(0), S.size(), 1);

113 // lp -= 0.5 \* dot\_product(S\_vec, W\_inv\_vec); // trace(S \* W^-1)

114  Eigen::Matrix<typename promote\_args<T\_y,T\_scale>::type,Eigen::Dynamic,Eigen::Dynamic> Winv\_S(mdivide\_left\_ldlt(ldlt\_W, static\_cast<Eigen::Matrix<T\_scale,Eigen::Dynamic,Eigen::Dynamic> >(S.template selfadjointView<Eigen::Lower>())));

115  lp -= 0.5\*trace(Winv\_S);

116  }

117  if (include\_summand<propto,T\_dof,T\_scale>::value)

118  lp += nu \* k \* NEG\_LOG\_TWO\_OVER\_TWO;

119  return lp;

120  }

121

122  template <typename T\_y, typename T\_dof, typename T\_scale>

123  inline

124  typename boost::math::tools::promote\_args<T\_y,T\_dof,T\_scale>::type

125  inv\_wishart\_log(const Eigen::Matrix<T\_y,Eigen::Dynamic,Eigen::Dynamic>& W,

126  const T\_dof& nu,

127  const Eigen::Matrix<T\_scale,Eigen::Dynamic,Eigen::Dynamic>& S) {

128  return inv\_wishart\_log<false>(W,nu,S);

129  }

130

131  template <class RNG>

132  inline Eigen::Matrix<double, Eigen::Dynamic, Eigen::Dynamic>

133  inv\_wishart\_rng(const double nu,

134  const Eigen::Matrix<double,Eigen::Dynamic,Eigen::Dynamic>& S,

135  RNG& rng) {

136

137  static const char\* function = "stan::prob::inv\_wishart\_rng(%1%)";

138

139  using stan::math::check\_greater;

140  using stan::math::check\_size\_match;

141  using Eigen::MatrixXd;

142  using stan::math::index\_type;

143

144  typename index\_type<MatrixXd>::type k = S.rows();

145

146  check\_greater(function, nu, k-1, "Degrees of freedom parameter",

147  (double\*)0);

148  check\_size\_match(function,

149  S.rows(), "Rows of scale parameter",

150  S.cols(), "columns of scale parameter", (double\*)0);

151

152  MatrixXd S\_inv = MatrixXd::Identity(k, k);

153  S\_inv = S.ldlt().solve(S\_inv);

154

155  return wishart\_rng(nu, S\_inv, rng).inverse();

156  }

157  }

158 }

159 #endif

matrix.hpp

mdivide\_left\_ldlt.hpp

stan::math::check\_greater

bool check\_greater(const char \*function, const T\_y &y, const T\_low &low, const char \*name, T\_result \*result)

**Definition:** check\_greater.hpp:58

traits.hpp

check\_size\_match.hpp

stan::math::check\_size\_match

bool check\_size\_match(const char \*function, T\_size1 i, const char \*name\_i, T\_size2 j, const char \*name\_j, T\_result \*result)

**Definition:** check\_size\_match.hpp:13

stan::math::mdivide\_left\_ldlt

Eigen::Matrix< typename boost::math::tools::promote\_args< T1, T2 >::type, R1, C2 > mdivide\_left\_ldlt(const stan::math::LDLT\_factor< T1, R1, C1 > &A, const Eigen::Matrix< T2, R2, C2 > &b)

Returns the solution of the system Ax=b given an LDLT\_factor of A.

**Definition:** mdivide\_left\_ldlt.hpp:24

stan::prob::inv\_wishart\_rng

Eigen::Matrix< double, Eigen::Dynamic, Eigen::Dynamic > inv\_wishart\_rng(const double nu, const Eigen::Matrix< double, Eigen::Dynamic, Eigen::Dynamic > &S, RNG &rng)

**Definition:** inv\_wishart.hpp:133

constants.hpp

traits.hpp

stan::agrad::mdivide\_left\_ldlt

Eigen::Matrix< fvar< T2 >, R1, C2 > mdivide\_left\_ldlt(const stan::math::LDLT\_factor< double, R1, C1 > &A, const Eigen::Matrix< fvar< T2 >, R2, C2 > &b)

Returns the solution of the system Ax=b given an LDLT\_factor of A.

**Definition:** mdivide\_left\_ldlt.hpp:25

check\_greater.hpp

stan::math::check\_ldlt\_factor

bool check\_ldlt\_factor(const char \*function, stan::math::LDLT\_factor< T, R, C > &A, const char \*name, T\_result \*result)

Return true if the underlying matrix is positive definite.

**Definition:** check\_ldlt\_factor.hpp:24

check\_ldlt\_factor.hpp

stan::math::index\_type

Primary template class for the metaprogram to compute the index type of a container.

**Definition:** index\_type.hpp:21

stan::prob::include\_summand

Template metaprogram to calculate whether a summand needs to be included in a proportional (log) prob...

**Definition:** traits.hpp:35

wishart.hpp

stan::math::lmgamma

boost::math::tools::promote\_args< T >::type lmgamma(const int k, T x)

Return the natural logarithm of the multivariate gamma function with the speciifed dimensions and arg...

**Definition:** lmgamma.hpp:57

rev.hpp

stan::math::LDLT\_factor

**Definition:** LDLT\_factor.hpp:16

log\_determinant\_ldlt.hpp

index\_type.hpp

stan::prob::inv\_wishart\_log

boost::math::tools::promote\_args< T\_y, T\_dof, T\_scale >::type inv\_wishart\_log(const Eigen::Matrix< T\_y, Eigen::Dynamic, Eigen::Dynamic > &W, const T\_dof &nu, const Eigen::Matrix< T\_scale, Eigen::Dynamic, Eigen::Dynamic > &S)

The log of the Inverse-Wishart density for the given W, degrees of freedom, and scale matrix...

**Definition:** inv\_wishart.hpp:52

stan::prob::wishart\_rng

Eigen::Matrix< double, Eigen::Dynamic, Eigen::Dynamic > wishart\_rng(const double nu, const Eigen::Matrix< double, Eigen::Dynamic, Eigen::Dynamic > &S, RNG &rng)

**Definition:** wishart.hpp:143

stan::agrad::log\_determinant\_ldlt

var log\_determinant\_ldlt(stan::math::LDLT\_factor< var, R, C > &A)

**Definition:** log\_determinant\_ldlt.hpp:48

stan::math::trace

T trace(const Eigen::Matrix< T, Eigen::Dynamic, Eigen::Dynamic > &m)

Returns the trace of the specified matrix.

**Definition:** trace.hpp:20

stan::math::log\_determinant\_ldlt

T log\_determinant\_ldlt(stan::math::LDLT\_factor< T, R, C > &A)

**Definition:** log\_determinant\_ldlt.hpp:12

stan::agrad::lmgamma

fvar< typename stan::return\_type< T, int >::type > lmgamma(int x1, const fvar< T > &x2)

**Definition:** lmgamma.hpp:16


---

[ Stan Home Page ]

*© 2011–2014,
Stan Development Team.*
